# Clinical outcome of radiofrequency ablation in patients with hepatocellular carcinoma aged 80 years and older

**DOI:** 10.1371/journal.pone.0334712

**Published:** 2025-10-14

**Authors:** Kenta Takaura, Kaoru Tsuchiya, Mina Taguchi, Yuzuki Tokunaga, Naoki Uchihara, Shohei Kimura, Yuuki Tanaka, Junko Yagita, Haruka Miyamoto, Taisei Keitoku, Risa Okada, Mayu Higuchi, Shohei Tanaka, Chiaki Maeyashiki, Nobuharu Tamaki, Yutaka Yasui, Yuka Takahashi, Hiroyuki Nakanishi, Namiki Izumi, Masayuki Kurosaki

**Affiliations:** Department of Gastroenterology and Hepatology, Musashino Red Cross Hospital, Tokyo, Japan; Al-Azhar University, EGYPT

## Abstract

**Background and aim:**

The incidence of hepatocellular carcinoma (HCC) is positively correlated with age, and the population of patients with HCC was also older at the time of clinical diagnosis. In the SURF trial, elderly people aged ≥80 years were excluded. We aimed to study the efficacy and safety of radiofrequency ablation (RFA) for patients aged over 80 years.

**Methods:**

Patients who underwent RFA at our institution for the initial treatment of HCC tumors with largest diameters of ≤3 cm, and ≤3 HCC nodules from January 2011 to December 2023. Treatment outcomes and prognoses were examined in the elderly group (≥80 years) and in the nonelderly group (<80 years). The Cox proportional hazards model was used to determine the factors associated with treatment outcomes and prognoses.

**Results:**

Of the 518 eligible patients, 136 patients were aged ≥80 years. The median overall survival (OS) values were 80 (95%CI; 60–96) and 123 (95%CI; 101–nucleotide analogs (NA)) months (p = 0.021) in the elderly and nonelderly groups, respectively. For liver disease-related deaths, the median OS values were 97 (95% CI; 80–NA) and NR (95% CI; NA–NA) months (p = 0.62) in the elderly and nonelderly groups, respectively. In the multivariate analysis, factors associated with OS were ALBI grade 2 or 3 (HR, 1.67, 95%CI; 1.07–2.60), DCP ≥ 40 mAU/ml (HR, 2.08, 95%CI; 1.42–3.04), persistent hepatitis C virus (HCV) infection (HR, 5.46, 95%CI; 3.08–9.69), and nonviral liver disease (HR, 4.19, 95%CI; 2.32–7.57).

The median recurrence-free survival values were 16 (95%CI; 14–22) and 26 (95%CI; 19–30) months, respectively (p = 0.023). HCC recurrence was significantly associated with the male sex (HR, 1.50, 95%CI; 1.17–1.93), elderly group (HR, 1.37, 95%CI; 1.10–1.95), ALBI grade 2 or 3 (HR, 1.39, 95%CI; 1.07–1.80), DCP ≥ 40 mAU/ml (HR, 1.41, 95%CI; 1.10–1.81), and persistent HCV infection (HR, 1.67, 95%CI; 1.30–2.15). The factors associated with liver disease-related death were ALBI grade 2 or 3 (HR, 2.17, 95% CI; 1.26–3.75), DCP ≥ 40 mAU/ml (HR, 2.33, 95% CI; 1.47–3.69), and persistent HCV infection (HR, 2.22, 95% CI; 1.39–3.56).

**Conclusions:**

In RFA for tumors with diameters of ≤3 cm and ≤3 HCC nodules, age over ≥80 years was not a significant factor associated with OS or liver disease-related death. The results support that RFA would be a promising treatment option for patients with HCC patients aged ≥80 years.

## Introduction

Liver cancer—including hepatocellular carcinoma (HCC)—remains the third-leading cause of death overall, the second-leading cause among men, and the fifth-leading cause among women worldwide [1]. In Asia (including Japan), liver cancer is the fifth most common malignancy and the second-leading cause of death, with Asia accounting for 72.5% of the world’s cases in 2020 [2]. HCC, the major histological type of primary liver cancer, accounts for approximately 75% of hepatic malignancies [3].

The incidence of HCC is positively correlated with age, and its prevalence is on the rise in Asia [2]. Similarly, the population of patients with HCC was also older at the time of clinical diagnosis in Japan [4]. The peak incidence rate of liver cancer by age group in Japan is ≥ 80 years [5]. These results suggest that opportunities to perform RFA in patients aged ≥80 years will increase in the future in the course of HCC treatment.

In the guidelines, treatment options for early-stage HCC within the Milan criteria (largest HCC diameter ≤3 cm and ≤3 HCC nodules) include radiofrequency ablation (RFA), hepatic resection, and liver transplantation [6–8]. RFA and hepatic resection remain the most common options in Japan. In the SURF trial—a randomized controlled trial comparing RFA and hepatic resection for HCC within the Milan criteria conducted in Japan—the recurrence-free survival was equivalent between the two treatment options [9]. However, in the SURF trial, elderly people aged ≥80 years were excluded. Therefore, the outcomes of RFA in patients aged ≥80 years are not clear. Considering the expected increase in the number of patients aged ≥80 years, it is important to evaluate the outcome of RFA treatment for this population in order to provide treatment options.

This study aimed to investigate the clinical outcome of early-stage HCC within the Milan criteria after RFA in elderly patients aged ≥80 years.

## Methods

This study was a retrospective study of patients who underwent RFA for HCC at Musashino Red Cross Hospital from January 2011 to December 2023. We included patients with early-stage HCC per the Milan criteria who underwent RFA as initial treatment. The elderly group was defined as those aged ≥80 years, and the nonelderly group as those aged under 80 years.

The diagnosis of HCC was made by histology and/or imaging with contrast-enhanced ultrasonography, dynamic CT, and/or Gd-EOB-DTPA-enhanced MRI (EOB-MRI) per the Japan Society of Hepatology (JSH) and American Association for the Study Liver Diseases guidelines [7,8]. When HCC is diagnosed and both surgical resection and RFA are possible, based on the patient’s choice and comorbidities, RFA was performed as the primary treatment for the patients. We use pethidine hydrochloride for pain management during treatment. After treatment, we administer naloxone hydrochloride, an antagonist, intravenously. As a rule, the efficacy of RFA was determined the day after treatment using dynamic CT or EOB-MRI according to the mRECIST criteria [10]. If there are obvious remnants of lesions on imaging, additional therapeutic interventions such as RFA, transcatheter arterial chemoembolization (TACE), hepatic resection, or stereotactic radiotherapy were performed at the discretion of the physician after considering tumor localization, liver function, and comorbidities.

Tumor markers such as alpha-fetoprotein (AFP) and des-γ-carboxy prothrombin (DCP) were used in the surveillance. Surveillance by imaging for post-RFA recurrence was performed every 3–6 months by dynamic CT, EOB-MRI, contrast-enhanced ultrasonography, or simple ultrasonography.

The data used in the study were age at first treatment, sex, aspartate aminotransferase (AST), alanine aminotransferase (ALT), platelet count (PLT), prothrombin time (PT), total bilirubin (T.Bil), albumin (Alb), the albumin-bilirubin (ALBI) grade, AFP, DCP, largest tumor diameter, number of tumors, and liver disease etiology (hepatitis B surface antigen [HBs Ag] positive or not, Anti-hepatitis C virus antibody [HCV Ab] positive or not, or both negative). The ALBI score was calculated using as follows:


ALBI score = (log10 bilirubin (μmol/L) × 0.66) + (albumin (g/L) × −0.085)

Based on the ALBI score, the patients were classified into three groups according to the ALBI grade: ALBI grade 1: ≤−2.60, 2: ≥−2.60 and <−1.39, and 3: ≥−1.39 [11]. For PT and DCP, patients taking warfarin or direct oral anticoagulants (DOACs) were treated as missing values.

For antiviral therapy, in patients with chronic hepatitis B, treatment was provided by nucleoside/nucleotide analogs (NA) at the time of chronic hepatitis diagnosis, liver cancer diagnosis, or after the first RFA. Moreover, in patients with chronic hepatitis C, we tried to achieve sustained viral response (SVR) with an interferon (IFN) regimen or an IFN-free regimen if there was no evidence of liver cancer at the time of chronic hepatitis diagnosis. After HCC treatment, we attempted to achieve SVR with an IFN regimen or an IFN-free regimen after confirming that there was no HCC recurrence within three months.

Follow-up was initiated at the time of the first RFA treatment, and overall survival, recurrence, and liver disease-related death were examined. Liver disease-related deaths were defined as deaths due to HCC or liver failure.

Written informed consent was obtained from all patients before the RFA procedure. The

requirement for written informed consent to be included in the study was waived because

of the retrospective design of the study. The privacy of all patients was fully protected. Information about this study was made available to the patients (posted on the hospital website), and the research subjects were given the opportunity to opt out. We accessed potentially personally identifiable medical data for research purposes from 30/12/2024–02/03/2025. This study was approved by the Ethics Committee of Musashino Red Cross Hospital and was conducted in accordance with the principles outlined in the Declaration of Helsinki.

### Statistical analysis

OS, recurrence-free survival, and liver disease-related death after HCC treatment were examined using the Kaplan–Meier method, and the difference in OS, recurrence-free survival, and liver disease-related death between the two groups was examined using the log-rank test. Factors associated with OS, recurrence, and liver disease-related death were examined using the Cox proportional hazards model. The threshold for statistical significance was set at p < 0.05, and all statistical analyses were performed using EZR [12].

## Results

### Baseline characteristics

The background information of this study’s 518 patients is shown in Table 1. There were 136 patients in the elderly group. The age at first treatment was 83 ± 3 years in the elderly group and 68 ± 9 years in the nonelderly group. Significant differences were observed in sex, ALT, PLT, PT, T.Bil, Alb, ALBI grade, and etiology between the two groups.

**Table 1:** Baseline Characteristics.

Characteristics	elderly group (n = 136)	non-elderly group (n = 382)	P value
Age(years old)	83 ± 3	68 ± 9	ー
Sex(male/female)	60/76	247/135	<0.001
AST(IU/L)	43 ± 31	46 ± 36	0.35
ALT(IU/L)	33 ± 28	40 ± 34	0.062
PLT(10⁴/μL)	14.1 ± 7.5	12.2 ± 5.6	0.002
PT(%)	93 ± 13	89 ± 16	0.007
T.Bil(mg/dL)	0.8 ± 0.4	1.0 ± 0.6	<0.001
Alb(g/dL)	3.6 ± 0.5	3.8 ± 0.6	0.015
ALBI grade(1/2/3)	37/95/4	155/208/19	0.008
AFP(ng/mL)	6.8(1.5-3151.9)	8.0(1.8-3510)	0.77
DCP(mAU/mL)	22.1(7.7-3069.6)	25.0(7.7-28700)	0.31
Largest tumor diameter(mm)	19 ± 5	18 ± 6	0.13
Number of tumors(1/2/3)	114/19/3	294/73/15	0.26
Etiology			<0.001
HBs Ag positive	3	50	
HCV Ab positive	91	210	
both negative	42	122	
			

AST, aspartate aminotransferase; ALT, alanine aminotransferase; PLT, platelets; PT, prothrombin time; T.Bil, total bilirubin; Alb, albumin; ALBI, albumin–bilirubin; AFP, alpha-fetoprotein; DCP, des- γ -carboxy-prothrombin; HBs Ag, hepatitis B virus surface antigen; HCV Ab, hepatitis C virus antibody; both negative, hepatitis B virus surface antigen negative and hepatitis C virus antibody negative

For HCV-infected participants, 34 out of 91 (37.4%) and 107 out of 210 (51.0%) patients in the elderly and nonelderly groups, respectively, achieved SVR before or after treatment. The remaining patients failed to achieve SVR during the observation period (persistent HCV infection).

### Overall survival, recurrence-free survival, and liver disease-related death

**Fig 1:**
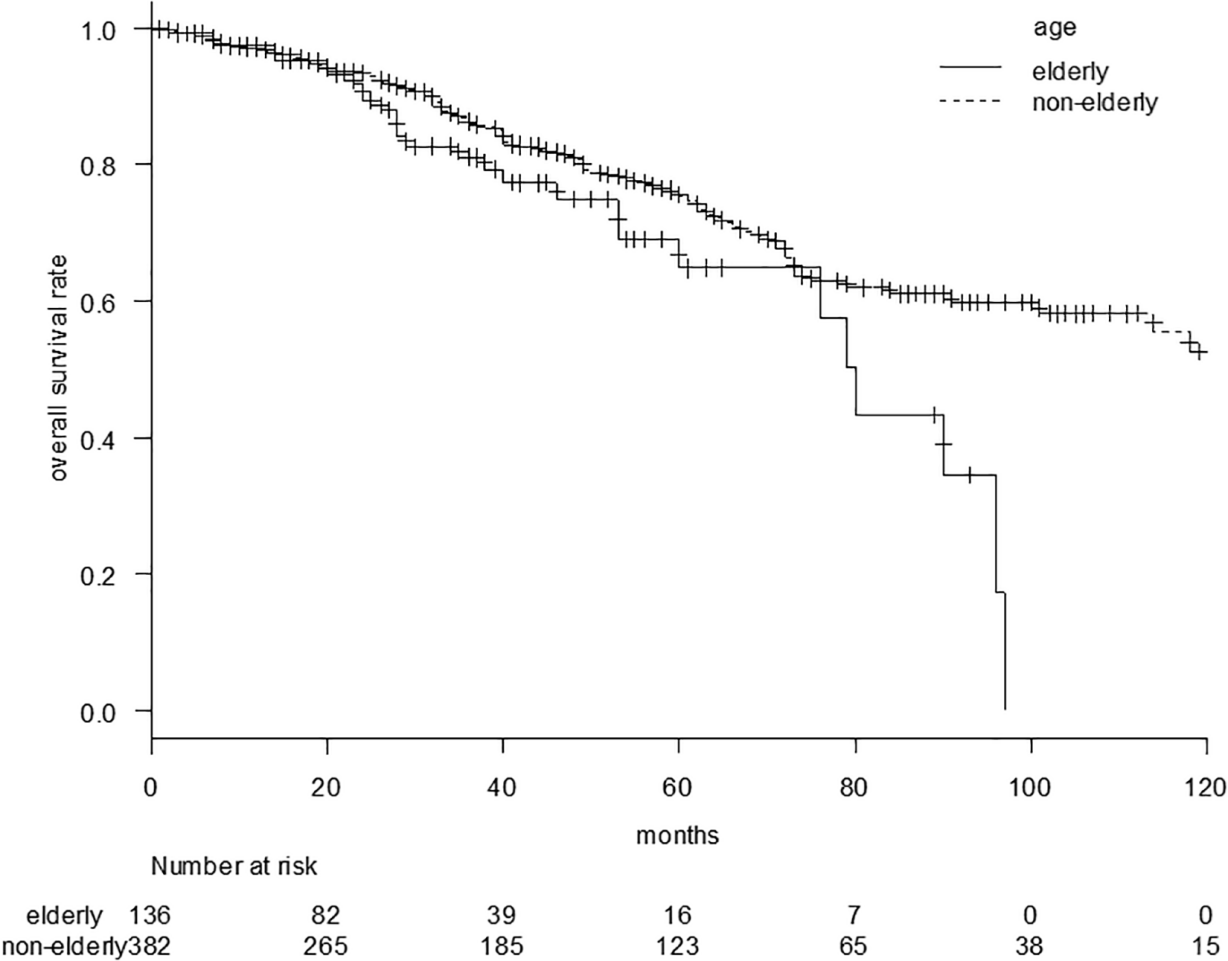
Overall survival curve comparing the elderly and nonelderly groups. The median overall survival (OS) was 80 (95%CI: 60–96) vs. 123 (95%CI: 101–NA) months in the elderly vs. nonelderly groups (p = 0.021).

The OS curves in elderly and nonelderly groups are shown in Fig 1. The observation period was 30 ± 24 months in the elderly group and 45 ± 35 months in the nonelderly group. The median overall survival (OS) was 80 (95% confidence interval [CI], 60–96) vs. 123 (95%CI, 101–NA) months in the elderly vs. nonelderly groups (p = 0.021). The survival rates at 3, 5, and 7 years were 81.1%, 64.8%, and 43.2% in the elderly group and 86.0%, 75.3%, and 61.0% in the nonelderly group, respectively.

**Fig 2:**
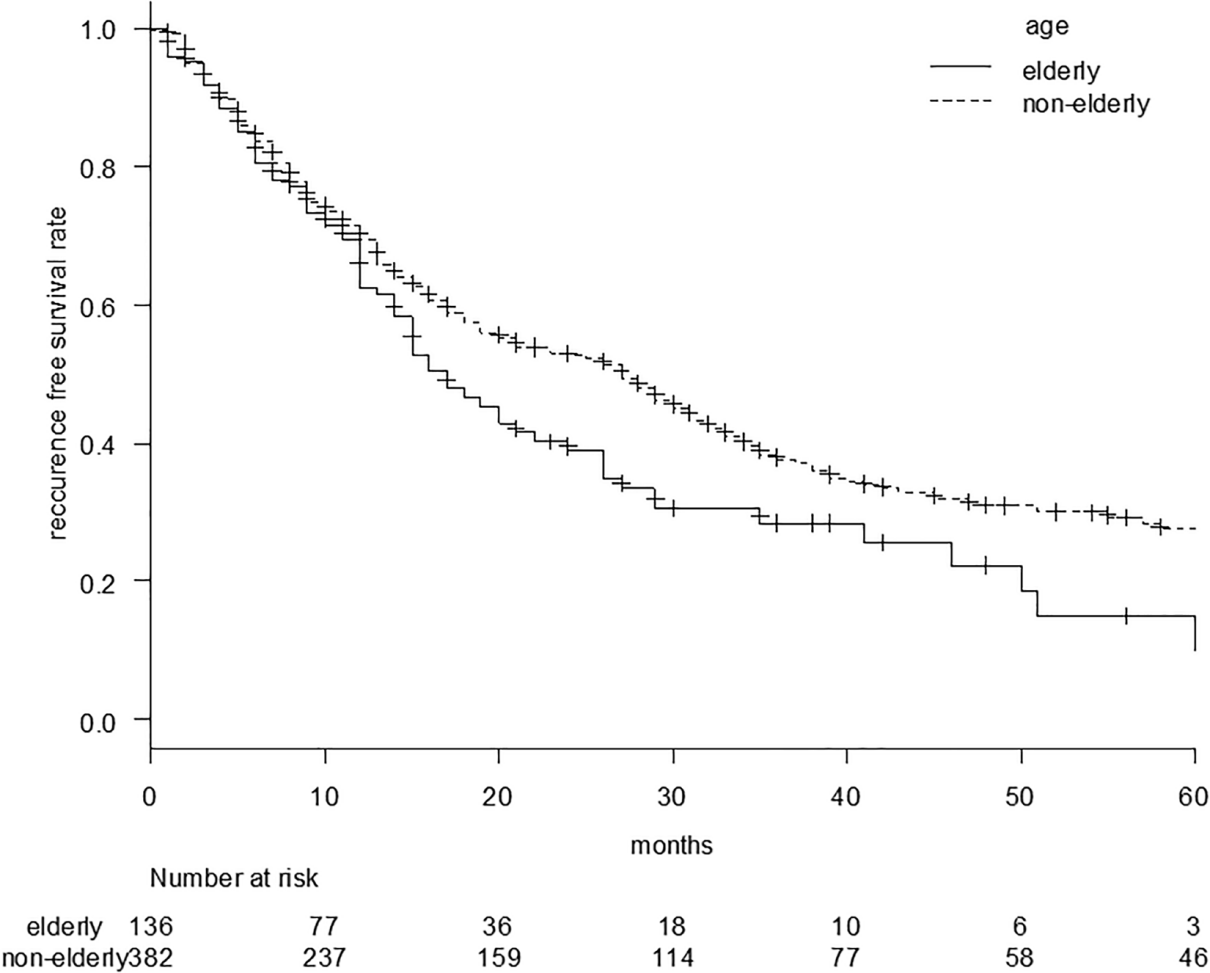
Recurrence-free survival curve comparing the elderly and nonelderly groups. The median recurrence-free survival was 17 (95%CI: 14–24) vs. 27 (95%CI: 20–31) months in the elderly vs. non-elderly groups (p = 0.017).

Similarly, the recurrence-free survival curve is shown in Fig 2. The median recurrence-free survival (RFS) was 17 (95%CI, 14–24) vs. 27 (95%CI, 20–31) months in the elderly vs. nonelderly groups (p = 0.017). The recurrence-free survival rates at 1 and 3 years were 62.5% and 28.4% in the elderly group and 69.4% and 37.5% in the nonelderly group, respectively.

**Fig 3:**
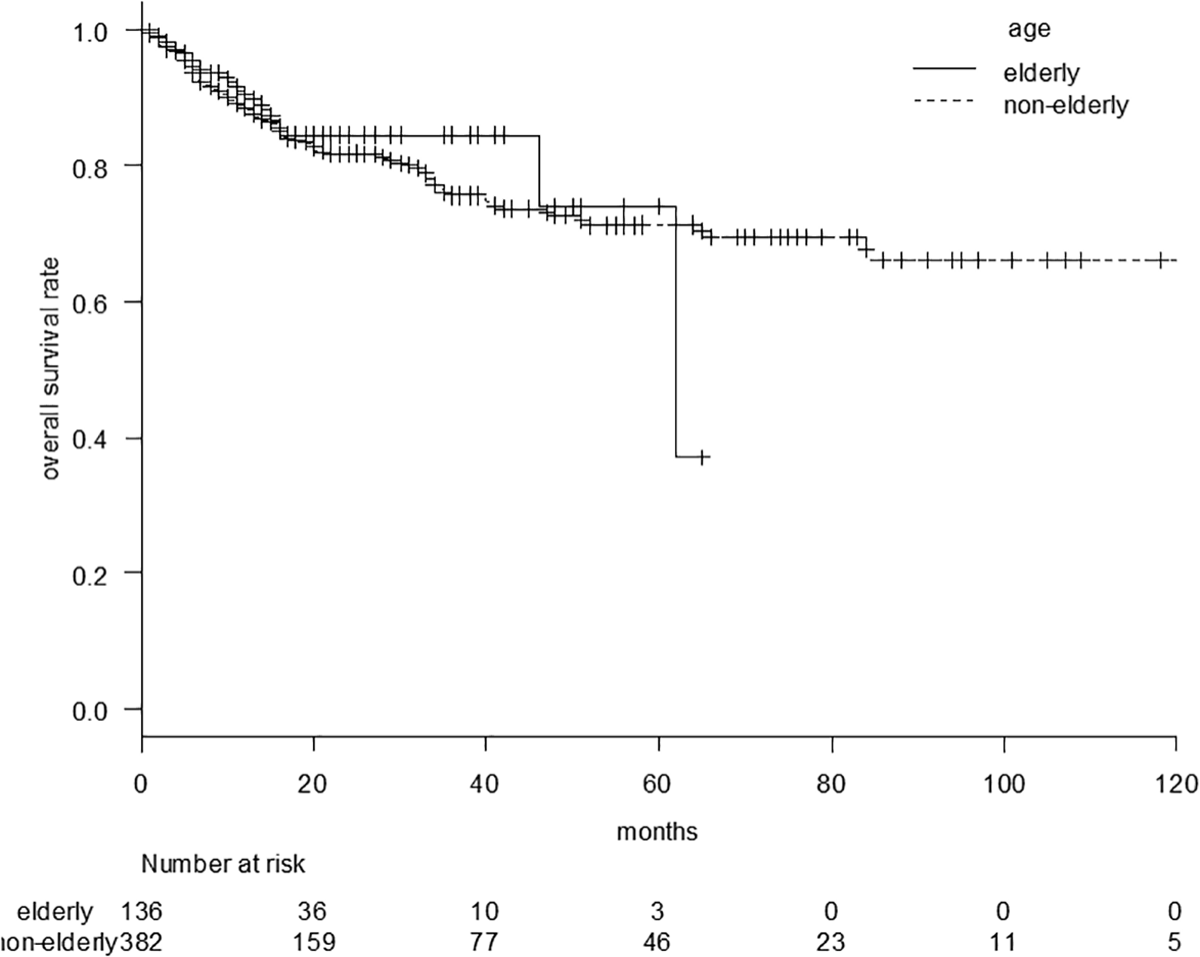
Overall survival curve for liver disease-related death comparing the elderly and nonelderly groups. The median overall survival for liver disease-related death was 97 (95%CI: 80–NA) months vs. NR in the elderly vs. nonelderly groups (p = 0.62).

The OS curves for liver disease-related death are shown in Fig 3. The median OS for liver disease-related death was 97 (95%CI, 80–NA) vs. NR months in the elderly vs. nonelderly groups (p = 0.62). The survival rates for liver disease-related death at 3, 5, and 7 years were 88.7%, 75.1%, and 64.3% in the elderly group and 89.6%, 80.1% and 67.7% in the nonelderly group, respectively.

Among all deaths, liver disease-related death occurred in 15 of 28 patients (53.6%) in the elderly group and 64 out of 85 patients (75.3%) in the nonelderly group. In the elderly group, 12 of these deaths were due to HCC and three were due to liver failure. In the nonelderly group, 37 of these deaths were due to HCC and 27 were due to liver failure.

On the other hand, the median OS for non-liver disease-related death was 96 (95%CI, 79–NA) vs. NR months in the elderly vs. nonelderly groups (p < 0.001). The survival rates for non-liver disease-related death at 3, 5, and 7 years were 91.5%, 86.4%, and 67.2% in the elderly group and 96.0%, 94.1%, and 90.1% in the nonelderly group, respectively.

### Factors associated with overall survival, recurrence-free survival, and liver disease-related death on Cox proportional hazards analyses

The results of our univariate and multivariate analyses of factors contributing to OS are shown in Table 2. Factors associated with OS were ALBI grade 2 or 3 (hazard ratio [HR], 1.67; 95% CI,1.07–2.60, p = 0.023), DCP ≥ 40 mAU/ml(HR, 2.08, 95% CI; 1.42–3.04, p < 0.001), persistent HCV infection (HR, 5.46, 95%CI; 3.08–9.69, p < 0.001) and nonviral liver disease (HR, 4.19, 95%CI; 2.32–7.57, p < 0.001).

**Table 2:** Factors related to overall survival on Cox proportional hazards analysis.

	Univariate analysis			Multivariate analysis		
Factors	HR	95%CI	P value	HR	95%CI	P value
Age(years old) ≤79 vs ≥ 80	1.67	1.08-2.59	0.022			
Sex female vs male	1.08	0.74-1.57	0.68			
AST(IU/L) ≤40 vs ≥ 41	1.6	1.10-2.31	0.013			
ALT(IU/L) ≤40 vs ≥ 41	0.84	0.57-1.25	0.4			
PLT(10⁴/μL) ≤9.9 vs ≥ 10	0.8	0.55-1.17	0.26			
PT(%) ≤80 vs ≥ 79.9	0.54	0.35-0.83	0.005			
ALBI grade 1 vs 2 or 3	2.13	1.41-3.21	<0.001	1.67	1.07-2.60	0.023
AFP(ng/mL) ≤19.9 vs ≥ 20	1.63	1.12-2.38	0.011			
DCP(mAU/ mL) ≤39.9 vs ≥ 40	2.10	1.44-3.06	<0.001	2.08	1.42-3.04	<0.001
Largest tumor diameter(mm) ≤19 vs ≥ 20	1.43	0.98-2.08	0.062			
Number of tumors(1/2/3) 1 vs 2 or 3	0.8	0.51-1.27	0.34			
HBV infection no vs yes	0.48	0.23-0.99	0.046			
Persistent HCV infection no vs yes	2.58	1.78-3.74	<0.001	5.46	3.08-9.69	<0.001
Nonviral liver disease no vs yes	1.55	1.05-2.28	0.026	4.19	2.32-7.57	<0.001

Similarly, factors associated with RFS are shown in Table 3. These factors included the elderly group (HR, 1.37, 95%CI; 1.10–1.95, p = 0.029), the male sex (HR, 1.50, 95%CI; 1.17–1.93, p = 0.001), ALBI grade 2 or 3 (HR, 1.39, 95%CI; 1.07–1.80, p = 0.012), DCP ≥ 40 mAU/ml (HR, 1.41, 95%CI; 1.10–1.81, p = 0.007), and persistent HCV infection (HR, 1.67, 95%CI; 1.30–2.15, p < 0.001).

**Table 3:** Factors related to recurrence free survival on Cox proportional hazards analysis.

	Univariate analysis			Multivariate analysis		
Foctors	HR	95%CI	P value	HR	95%CI	P value
Age(years old) ≤79 vs ≥ 80	1.38	1.06-1.80	0.019	1.37	1.03-1.83	0.029
Sex female vs male	1.38	1.09-1.74	0.007	1.50	1.17-1.93	0.001
AST(IU/L) ≤40 vs ≥ 41	1.26	1.00-1.58	0.051			
ALT(IU/L) ≤40 vs ≥ 41	0.96	0.75-1.22	0.73			
PLT(10⁴/μL) ≤9.9 vs ≥ 10	0.86	0.68-1.09	0.22			
PT(%) ≤80 vs ≥ 79.9	0.7	0.54-0.92	0.009			
ALBI grade 1 vs 2 or 3	1.59	1.25-2.03	<0.001	1.39	1.07-1.80	0.012
AFP(ng/mL) ≤19.9 vs ≥ 80	1.43	1.12-1.81	0.004			
DCP(mAU/mL) ≤39.9 vs ≥ 40	1.48	1.16-1.89	0.002	1.41	1.10-1.81	0.007
Largest tumor diameter(mm) ≤19 vs ≥ 20	1.45	1.15-1.83	0.002			
Number of tumors(1/2/3) 1 vs 2 or 3	1.41	1.08-1.84	0.011			
HBV infection no vs yes	0.85	0.58-1.24	0.4			
Persistent HCV infection no vs yes	1.80	1.42-2.28	<0.001	1.67	1.30-2.15	<0.001
Nonviral liver disease no vs yes	0.98	0.76-1.26	0.87			

Moreover, factors associated with liver disease-related death are shown in Table 4. These factors include ALBI grade 2 or 3 (HR, 2.17, 95% CI; 1.26–3.75, p = 0.006), DCP ≥ 40 mAU/ml (HR, 2.33, 95% CI; 1.47–3.69, p < 0.001) and persistent HCV infection (HR, 2.22, 95% CI; 1.39–3.56, p < 0.001).

In OS, RFS, and liver disease-related death, ALBI grade 2 or 3, DCP ≥ 40 mAU/ml and persistent HCV infection were identified as significant prognostic factors.

**Table 4:** Factors related to liver disease-related death on Cox proportional hazards analysis.

	Univariate analysis			Multivariate analysis		
Factors	HR	95%CI	P value	HR	95%CI	P value
Age(years old) ≤79 vs ≥ 80	1.16	0.65-2.05	0.62			
Sex female vs male	1.49	0.94-2.38	0.092			
AST(IU/L) ≤40 vs ≥ 41	1.65	1.06-2.57	0.023			
ALT(IU/L) ≤40 vs ≥ 41	0.85	0.53-1.37	0.50			
PLT(10⁴/μL) ≤9.9 vs ≥ 10	0.63	0.40-0.98	0.039			
PT(%) ≤80 vs ≥ 79.9	0.5	0.30-0.83	0.007			
ALBI grade 1 ve 2 or 3	2.45	1.51-4.14	<0.001	2.17	1.26-3.75	0.006
AFP(ng/mL) ≤19.9 vs ≥ 20	1.86	1.19-2.90	0.006			
DCP(mAU/mL) ≤39.9 vs ≥ 40	2.22	1.41-3.48	<0.001	2.33	1.47-3.69	<0.001
Largest tumor diameter(mm) ≤19 vs ≥ 20	1.73	1.11-2.70	0.015			
Number of tumors(1/2/3) 1 vs 2 or 3	0.93	0.55-1.59	0.81			
HBV infection no vs yes	0.73	0.35-1.53	0.41			
Persistent HCV infection no vs yes	2.33	1.50-3.63	<0.001	2.22	1.39-3.56	<0.001
Nonviral liver disease no vs yes	1.56	0.98-2.47	0.061			

### Complications associated with RFA

Twenty of the 518 patients (3.9%) had treatment-related complications in all patients.

In the elderly group, four cases involved bleeding from the puncture site, and one of four required transcatheter arterial embolization. One patient showed deterioration of physical function and required transfer to a hospital for rehabilitation. Pneumothorax was observed in two patients, renal dysfunction in one, portal vein thrombosis in one, and intrahepatic blood flow disorder in one, all of which were self-limiting.

In the nonelderly group, three cases involved bleeding from the puncture site, and two of these three required transcatheter arterial embolization. Symptoms suggestive of gastrointestinal bleeding were observed in two patients; however, the bleeding sites could not be identified from endoscopic or imaging findings. Three patients had renal dysfunction, one had suspected gastrointestinal perforation, and one had suspected cholecystitis, all of which resolved spontaneously.

No treatment-related deaths were observed in either group

## Discussion

RFA is one of the effective treatment options for HCC because of its high efficacy and low frequency of complications [13,14]. Previous reports on the prognosis of patients treated using RFA for elderly HCC patients, although aged ≥75 years, showed that the 3- and 5-year survival rates were 64.1–82.5% and 49.1–61.0%, respectively [15–17]. In our study, despite focusing on patients aged ≥80 years, the 3-year and 5-year survival rates were similar to those in previous reports.

Like RFA, resection is one of the recommended treatment options in the local treatment of HCC. In several studies comparing RFA with resection for HCC, the latter has a better prognosis but a higher complication rate and a longer hospitalization period [18–21]. Especially in elderly patients, resection may lead to higher complication and in-hospital mortality rates [22]. In this respect, RFA is a better option for the treatment of HCC in elderly patients.

DCP ≥ 40 mAU/ml is one of the prognostic factors in this study. DCP is known to be one of the diagnostic and prognostic markers of HCC [23–27]. High serum DCP levels reflect the biological aggressiveness and progression of HCC tumors, such as microvascular invasion, which cannot be detected on imaging [28,29]. In this regard, high serum DCP is a poor prognostic factor; therefore, treatment options other than RFA or careful follow-up after treatment should be considered.

Persistent HCV infection is also one of the prognostic factors in this study. As for HCV, SVR can be achieved at a high rate with only a few side effects when DAA remedies are used [30–33]. In terms of HCC treatment, achieving SVR reportedly improves OS and RFS regardless of whether the patient is treated before or after HCC [34–36]. Moreover, some studies have reported that HCV treatment also improves liver function and contributes to the improvement of OS by reducing the risk of non-liver diseases such as cardiovascular and neurological diseases [36–39]. As such, it is important to achieve SVR during HCC treatment.

In addition, nonviral liver disease is also one of the prognostic factors. As previously reported, HCC with a background of nonviral liver disease has been shown to have a poorer prognosis than viral liver disease [40]. Most nonviral liver diseases are steatotic liver disease (SLD) including alcohol-associated (-related) liver disease and metabolic dysfunction-associated steatohepatitis. At least, in our study, 112 of 164 (68.3%) cases are SLD. These liver diseases can also be difficult to treat, and like persistent HCV infection, they may affect prognosis. Further research is needed on this point.

In this study, being an elderly patient was one of the risk factors for recurrence. In a previous study, elderly patients had more frequent local progression [17]. However, age was not a risk factor for OS or liver disease-related death in our study, so appropriate treatment after recurrence is important for the patient’s prognosis. RFA is also reported as an effective treatment option for recurrent HCC [41]. We expect RFA to become an important treatment option in the treatment of recurrent HCC in elderly patients.

Nevertheless, this study has several limitations. A primary limitation of this study was its single-center, retrospective design that did not consider the concomitant bias, including the time bias of when SVR was achieved and the differences in devices used based on the time of RFA implementation. Second, most of the tumors were diagnosed only via imaging. Therefore, we were not able to evaluate histological differentiation and vascular invasion, which is one of the prognostic factors in this study. However, in this study, the median maximum tumor diameter was less than 20 mm in both groups. It was difficult to collect all tumor tissues before treatment. Third, differences in tumor location were not considered. In a previous study, tumor location—such as periportal-vein or perihepatic vein—was an important determinant of RFA efficacy [42]. This difference may have affected the difference in RFS.

In conclusion, being ≥80 years was not significantly associated with OS or liver disease-related death for HCC within the Milan criteria. Per our findings, RFA is a promising treatment option for patients with HCC aged ≥80 years.
